# The Best Predictor of Future Behavior May Be the Past: Exploring Behavior Change in Men Who Have Sex with Men Using Pre-exposure Prophylaxis in the Netherlands

**DOI:** 10.1007/s10508-024-02863-z

**Published:** 2024-05-06

**Authors:** Daphne van Wees, Liza Coyer, Mark van den Elshout, Eline Op de Coul, Fleur van Aar

**Affiliations:** 1 Center for Infectious Disease Control, National Institute for Public Health and the Environment (RIVM), P.O Box 1, 3720 BA Bilthoven, The Netherlands; 2 Department of Infectious Diseases, Research and Prevention, Public Health Service of Amsterdam, Amsterdam, The Netherlands; 3 Municipal Health Service for the Utrecht Region, Utrecht, The Netherlands

**Keywords:** Sexual behavior, Pre-exposure prophylaxis, Men who have sex with men, Sexually transmitted infections, Sexual orientation

## Abstract

Pre-exposure prophylaxis (PrEP) use prevents HIV transmission, and may lead to changes in sexual behavior. We aimed to explore sexual behavior over time, and identify predictors of behavior change in men who have sex with men (MSM) using PrEP at sexual health centers (SHC) in the Netherlands. We used longitudinal data from the national STI surveillance database (January 2018–June 2021) of HIV-negative MSM who first initiated PrEP in the national PrEP pilot. We modelled behavior change after PrEP initiation, and identified predictors of behavior change using multi-state Markov models. The probability of stopping group sex, chemsex, use of poppers or erection stimulants, and having ≥ 10 partners in the past 6 months among included MSM (n = 4,349, n SHC visits = 21,820) was higher compared to the probability of starting with these behaviors after PrEP initiation. However, MSM who used condoms consistently during anal sex had a high probability of changing to inconsistent condom use (0.8) at the next visit, and inconsistent condom users mostly remained inconsistent (0.8). First visit, visiting the SHC more often (vs. regular), and STI diagnosis were predictors of starting or continuing with most of the behaviors associated with increased risk. Behavior change was less likely among older participants (> 34 compared to ≤ 34 years), and during COVID-19 lockdown and post-lockdown periods compared to pre-COVID-19. Although condom use decreased over time, transitions towards stopping with other behaviors associated with increased likelihood of acquiring an STI after PrEP initiation were common. This may suggest increased sexual empowerment, especially among younger MSM. Predictors of behavior change may help to identify MSM who are likely to start with or continue to engage in these behaviors in the near future and to provide suitable and timely counselling about behavior and PrEP adherence.

**Supplementary Information:**

The online version contains supplementary material available at 10.1007/s10508-024-02863-z.

## Introduction

Pre-exposure Prophylaxis (PrEP) is a highly effective combination of drugs that can be used to prevent HIV. Since the implementation of a 5-year national PrEP pilot program in 2019 in the Netherlands, sexual health centers (SHCs) have been providing PrEP and PrEP care to individuals with an increased likelihood to acquire HIV (Hoornenborg et al., 2018a, 2018b; van Wees et al., 2022). The Dutch national guideline indicates PrEP specifically for men who have sex with men (MSM) and transgender persons who have had condomless insertive or receptive anal sex with a male or transgender partner with an unknown HIV status, or with a partner known to live with HIV with a detectable viral load, an anal sexually transmitted infection (STI), syphilis, or used post-exposure prophylaxis (PEP) in the past 6 months (Bierman et al., 2022).

Many countries have implemented PrEP programs and the need for closely monitoring of STI/HIV incidence and behavior among PrEP users in these programs has been underlined previously (van Wees et al., 2022). Concerns have been raised that PrEP use may lead to behavior change (Sarno et al., 2021). For instance, as the likelihood of acquiring HIV substantially decreases as a consequence of PrEP use (Rozhnova et al., 2018), previous studies have shown that PrEP users reduced using other HIV risk reduction strategies, such as condom use (Hoornenborg et al., 2018a, 2018b, 2019; Prestage et al., 2019; Traeger et al., 2018). This may lead to increased incidences of other STIs, such as chlamydia, gonorrhea, and syphilis (van Bilsen et al., 2020; Walker et al., 2022). Previous research has mainly focused on condom use among PrEP users (Hoornenborg et al., 2018a, 2018b, 2019; Prestage et al., 2019; Traeger et al., 2018), since this is directly associated with an increased likelihood of STI/HIV. However, there are other key behaviors associated with inconsistent condom use and with an increased likelihood of STI/HIV, such as a high number of sexual partners (e.g., ≥ 10 partners in past 6 months) (Armstrong et al., 2018; Basten et al., 2021; Smith et al., 2012), use of poppers (Basten et al., 2021; Smith et al., 2012), chemsex (e.g., drug use before or during sex, including injecting drug use (slamming)) (De Baetselier et al., 2021; Flores Anato et al., 2022; Knoops et al., 2021; Maxwell et al., 2019; Vosburgh et al., 2012), and group sex (Basten et al., 2021; Grov et al., 2013; Knox et al., 2020; Prestage et al., 2009). Furthermore, previous studies have identified factors that may influence these behaviors, such as age, previous STI diagnoses (Basten et al., 2021) and COVID-19 prevention measures (Jongen et al., 2021a, 2021b; van Bilsen et al., 2021). However, participants in these previous studies were included before the start of the national PrEP pilot program (i.e., PrEP was not yet widely available) or were recruited through PrEP demonstration and effectiveness studies (i.e., different population of PrEP users). Thus, research into PrEP use and behavior change on a national level is lacking. Longitudinal analyses of sexual behavior —and factors that may influence these behaviors—among PrEP users on a national level might provide additional insights into the impact of PrEP use on STI incidence, and may be used to inform prevention strategies and behavior change interventions.

The aim of this study was to examine behavior change among MSM who first initiated PrEP use in the national PrEP pilot in the Netherlands. Furthermore, we aimed to identify predictors of behavior change. We used longitudinal data from all SHCs in the Netherlands between January 2018 and June 2021 as reported to the national surveillance database of the National Institute for Public Health and the Environment (RIVM).

## Method

### Participants

In the Netherlands, SHCs offer free-of-charge STI and HIV testing to people with an increased likelihood of acquiring STIs, such as MSM. Additionally, SHCs have been carrying out a national PrEP pilot program since August 2019*.* The pilot has a limited financial budget, and a maximum of 8500 individuals can participate nationally. Furthermore, the maximum number of individuals that can be included in the pilot is different in each SHC region.

During 3-monthly follow-up consultations, PrEP pilot participants were routinely tested free-of-charge for chlamydia, gonorrhea, syphilis, and HIV, and for lymphogranuloma venereum (LGV), hepatitis B, and hepatitis C on indication (e.g., notified by partner, symptoms). Furthermore, PrEP pills (€7, 50 a month) and other necessary care was provided, and sexual behavior was carefully monitored at these consultations (Hoornenborg & Rijnders, 2019). Participants in the PrEP pilot may have also visited the SHC for additional STI/HIV test consultations between PrEP follow-up consultations, for example in case of STI-related symptoms or when notified of STI exposure.

### Procedure and Measures

All regular STI/HIV test consultations, PrEP start, and follow-up consultations were routinely registered by the SHCs in their electronic patient file. SHCs send a predefined selection of information from the patient files to the national database for surveillance purposes. SHC visitors either provided verbal informed consent for sharing data with the RIVM or used an opt-out option. Registered national data are pseudonymised and secured in accordance with European privacy legislation. In the current study, we used longitudinal data between January 1, 2018 and June 30, 2021 from the national surveillance database. Multiple consultations of a unique individual could be linked using an identification number of the electronic patient file.

Demographic characteristics included age (categories based on median age at pilot program entry), sex of sexual partners (MSM vs men who have sex with men and women [MSMW]), education: Category 1: no education, primary education only, and the various pathways of prevocational secondary education (VMBO) including lower secondary vocational training and assistant’s training (MBO-1); Category 2: upper secondary education (HAVO/VWO), basic vocational training (MBO-2), vocational training (MBO-3), and middle management and specialist education (MBO-4); Category 3: associate degree programmes, higher education (HBO/WO) Bachelor programmes; 4-year education at universities of applied sciences (HBO); Master degree programmes at universities of applied sciences and at research universities (HBO, WO); and doctoral degree programmes at research universities (WO), and region of origin. Region of origin is based on the individuals’ and the individuals’ parents’ country of birth. First, we classified persons as born in the Netherlands, migrants, or children of migrants (Statistics Netherlands (CBS), 2022). Migrants includes persons who are born abroad, and children of migrants are persons who are born in the Netherlands and have at least one parent who is born abroad. Then, we further divided migrants and children of migrants into regions included in the SHC triage indication (i.e., Turkey, Morocco, Suriname, CAS-BES islands, Indonesia, Eastern Europe, Africa, Latin America, and Asia) or migrants from other regions.

Behavioral data included group sex (yes/no), chemsex (yes/no) defined as the use of one or more of the following drugs before or during sex: crystal meth, mephedrone or gamma-hydroxybutyric acid/gamma-butyrolactone (GHB/GBL), use of poppers or erection stimulants (yes/no), partner numbers (categorized for analysis and implementation purposes, based on percentiles in data: ≤ 25th percentile, 25th–75th percentile, and ≥ 75th percentile), and receptive and insertive anal sex (no anal sex, anal sex with consistent condom use, anal sex with inconsistent condom use), all in the past 6 months.

Additional data from the consultations included SHC region of current visit (Amsterdam vs non-Amsterdam), PrEP use (no/yes, 4–12 months ago/yes, in the past 3 months), PrEP regimen (daily/intermittent/both), type of consultation (regular STI/HIV test consultation/PrEP start consultation/PrEP follow-up consultation), PEP (yes/no), and STI/HIV test results, including gonorrhea and chlamydia at one or multiple anatomical locations (urethral, anorectal, oral), LGV, syphilis, HIV, hepatitis B, hepatitis C tests and diagnoses, if relevant. We defined time between consultations as “visits regularly” (i.e., 5–7 months between regular consultations, and 2–4 months between PrEP consultations), “visits more often” (i.e., < 5 months between regular consultations, and < 2 months between PrEP consultations), “visits less often” (i.e., > 7 months between regular consultations, and > 4 months between PrEP consultations), and first visit (i.e., first SHC visit ever or first visit within the study period). We classified timing of visits as pre-COVID-19 (i.e., January 2018–March 14, 2020), COVID-19 lockdown (i.e., March 15, 2020–May 31, 2020 and December 1, 2020–June 30, 2021), and COVID-19 post-lockdown (i.e., June 1, 2020–November 30, 2020) (Rijksoverheid, 2021).

### Statistical Analysis

All HIV-negative MSM and MSMW who first initiated PrEP use in the national PrEP pilot (i.e., never used PrEP before or reported initiating PrEP use recently at the first PrEP consultation) and who had had at least two SHC visits in the study period were included in the statistical analyses. Transgender or gender diverse persons (due to small number of participants in the PrEP pilot (van Wees et al., 2022)), individuals who never used PrEP, individuals who had used PrEP before the PrEP pilot started, and individuals who were already using PrEP via another provider (e.g., general practitioner, HIV physician, PrEP study, informal routes) were excluded. Follow-up for each individual started at the last SHC visit before the PrEP start consultation (earliest January 1, 2018) or the PrEP start consultation (i.e., individuals had no SHC visits before the PrEP start consultation) and ended at the first HIV seroconversion visit or the last visit before July 1, 2021. Thus, our dataset included regular STI/HIV consultations, PrEP start consultations, and PrEP follow-up consultations.

We modelled behavior change over time using multistate time-homogenous Markov models (Kapland, 2008). These models are well-suited to analyze longitudinal data with uneven follow-up time (i.e., 3-monthly visits in the PrEP pilot program, but also visits in between). Furthermore, based on all follow-up data, these models allow for identification of predictors of behavior change (i.e., information at current visit may predict outcome at next visit).

For each behavioral outcome, we constructed a separate model, and we modelled transition intensities between different categories of the behavioral outcomes. These transitions included starting or stopping group sex, chemsex, and use of poppers or erection stimulants, changing partner numbers, and changing between no receptive or insertive anal sex, receptive or insertive anal sex with consistent condom use, and receptive or insertive with inconsistent condom use. As the number of visits is not the same for each individual, we used maximum likelihood estimation as a method to account for these differences in follow-up time. These transition intensities represent the likelihood of changing behavior at time t + 1, given the behavior reported at time t. The resulting transition intensities are mean transition probabilities for each behavioral outcome in the study period between January 2018 and June 2021. If the outcome was missing, we excluded the visit from the model. Furthermore, if HIV was diagnosed, this visit was included as an absorbent state in the model (i.e., transition intensity is zero). We assessed goodness of fit for the final model by comparing values of the Akaike Information Criterion (AIC, lower values indicating a better fit) for simpler models (i.e., less predictors to check for overfitting) and for different Q-matrices (representing the intensities of transitions between states).

We also examined age, SHC region, anal STI (chlamydia or gonorrhea) or syphilis, non-anal STI (chlamydia or gonorrhea), time between consultations, and COVID-19 as potential predictors of behavior change. To determine whether potential predictors reported at time t were significantly associated with behavior change (i.e., transitioning) at the next visit, we calculated hazard ratios (HR) and 95% confidence intervals (CIs) for each potential predictor in univariable and multivariable models for each behavioral outcome separately. All the predictors included in the univariable models were included in the multivariable models, irrespective of statistical significance of the variable in the univariable model. When potential predictors were highly correlated with each other, the strongest predictor was included in the model based on the hazard ratios. When the number of observations in specific transitions was too small (< 5%), the potential predictor was not included in the model. Data management and statistical analysis were done in R version 4.2.0 (R Development Core Team, 2020), and the Markov models were estimated using the ‘msm’ package in R (Jackson, 2011).

## Results

### Sample Characteristics

**Fig. 1:**
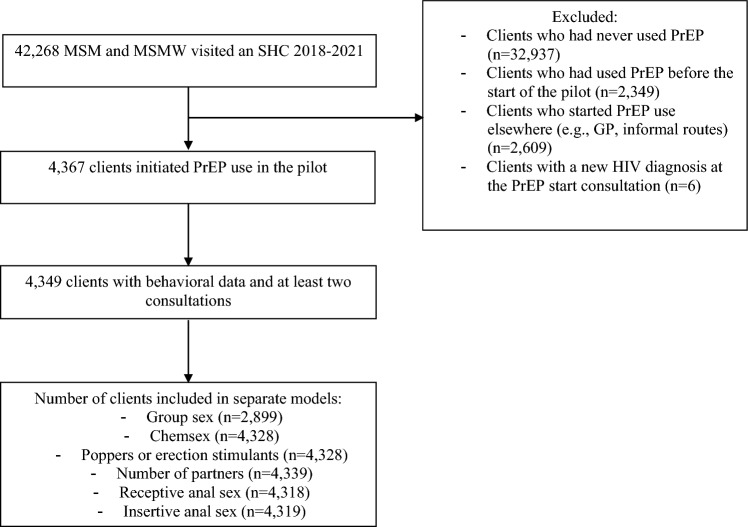
Flowchart of clients included in the study who initiated PrEP use in the national PrEP pilot and who had at least two consultations at an SHC between January 2018 and June 2021. Chemsex was defined as the use of (a combination of) crystal meth, mephedrone or gamma-hydroxybutyric acid/gamma-butyrolactone (GHB/GBL) before or during sex. GP = General Practitioner; HIV = Human Immunodeficiency Virus; MSM = Men Who Have Sex with Men; MSMW = Men Who Have Sex with Men and Women; PrEP = Pre-Exposure Prophylaxis

A total of 42,268 MSM and MSMW visited the SHC between January 2018 and June 2021. Of these individuals, 4367 initiated PrEP use for the first time in the national PrEP pilot (Fig. 1). The majority of individuals included in the study was ≤ 34 years old (vs > 34 years old), had sex with men only, were currently enrolled or finished university, and were born in the Netherlands (Table 1). Of these individuals, 4349 had had at least two visits with behavioral data in the study period (i.e., one pre-PrEP consultation and ≥ 1 PrEP consultation or ≥ 2 PrEP consultations), and a total of 21,820 study visits were included in the analysis. For 80% of all participants, an SHC visit was available before the PrEP start consultation, and the mean number of months between this “pre-PrEP” visit and the PrEP start consultation was 4 months (minimum 1 week and maximum 40 months). At the pre-PrEP consultation or if this was not available, PrEP start consultation, group sex (21%), chemsex (20%), poppers or erection stimulants (24%), ≥ 10 partners (36%), and inconsistent condom use during receptive (78%) and insertive (71%) anal sex in the past 6 months were commonly reported.

**Table 1:** Characteristics at the first visit of men who have sex with men who visited the SHC between January 2018 and June 2021, and initiated PrEP use in the national PrEP pilot

	n = 4367 n visits = 21820
	n (%)
Age at first visit	
≤ 34 years	2462 (56)
> 34 years	1905 (44)
Sexual orientation	
MSM	4008 (92)
MSMW	365 (8)
Education level first visit^a^	
Category 1	430 (10)
Category 2	952 (22)
Category 3	2606 (60)
Region of origin^b^	
Netherlands	2532 (58)
Migrant with triage indication	989 (23)
Child of migrant with triage indication	354 (8)
Other migrant	491 (11)

^a^Category 1: no education, elementary school, lbo, mavo, vmbo, mbo-1; Category 2: mbo 2–4, havo, vwo; Category 3: university of applied sciences, university

^b^Regions of origin with triage indication include Turkey, Morocco, Suriname, CAS-BES islands, Indonesia, Eastern Europe, Africa, Latin America, and Asia

Categories do not always add up to 100%, as missings are not shown

*MSM* Men Who Have Sex with Men *MSMW* Men Who Have Sex with Men and Women *SHC* Sexual Health Center

Longitudinal description of characteristics are provided in Table 2. Mean follow-up time was 17 months (median = 17, IQR = 7–25 months), and median number of consultations was 4 (minimum 2 and maximum 22). Furthermore, at 21% of all visits an STI was diagnosed, of which most were gonorrhea (positivity = 12%) or chlamydia (positivity = 11%) and some were syphilis infections (positivity = 2%). In addition, 102 LGV diagnoses, 5 infectious hepatitis B diagnoses, 7 infectious hepatitis C diagnoses, and 3 new HIV diagnoses were registered.

**Table 2:** Number of visits by potential predictor of behavior change between January 2018 and June 2021

	n (%)
Total	21,820
Age	
≤ 34 years	11,222 (51)
> 34 years	10,598 (49)
SHC region,	
Amsterdam	8,290 (38)
Non-Amsterdam	13,530 (62)
Anal STI or syphilis diagnosis current visit	
Yes	3634 (17)
No	18186 (83)
Non-anal STI diagnosis current visit	
Yes	1207 (6)
No	20,605 (94)
Time between consultations	
Regular	11094 (51)
More often	3391 (16)
Less often	2734 (13)
First visit	4367 (20)
COVID-19	
Pre-COVID-19	6104 (28)
Lockdown	9855 (45)
Post-lockdown	5861 (27)

Numbers do not always add up to total number of visits, as missings are not shown

*STI* Sexually Transmitted Infection

### Behavior Change Over Time

**Fig. 2:**
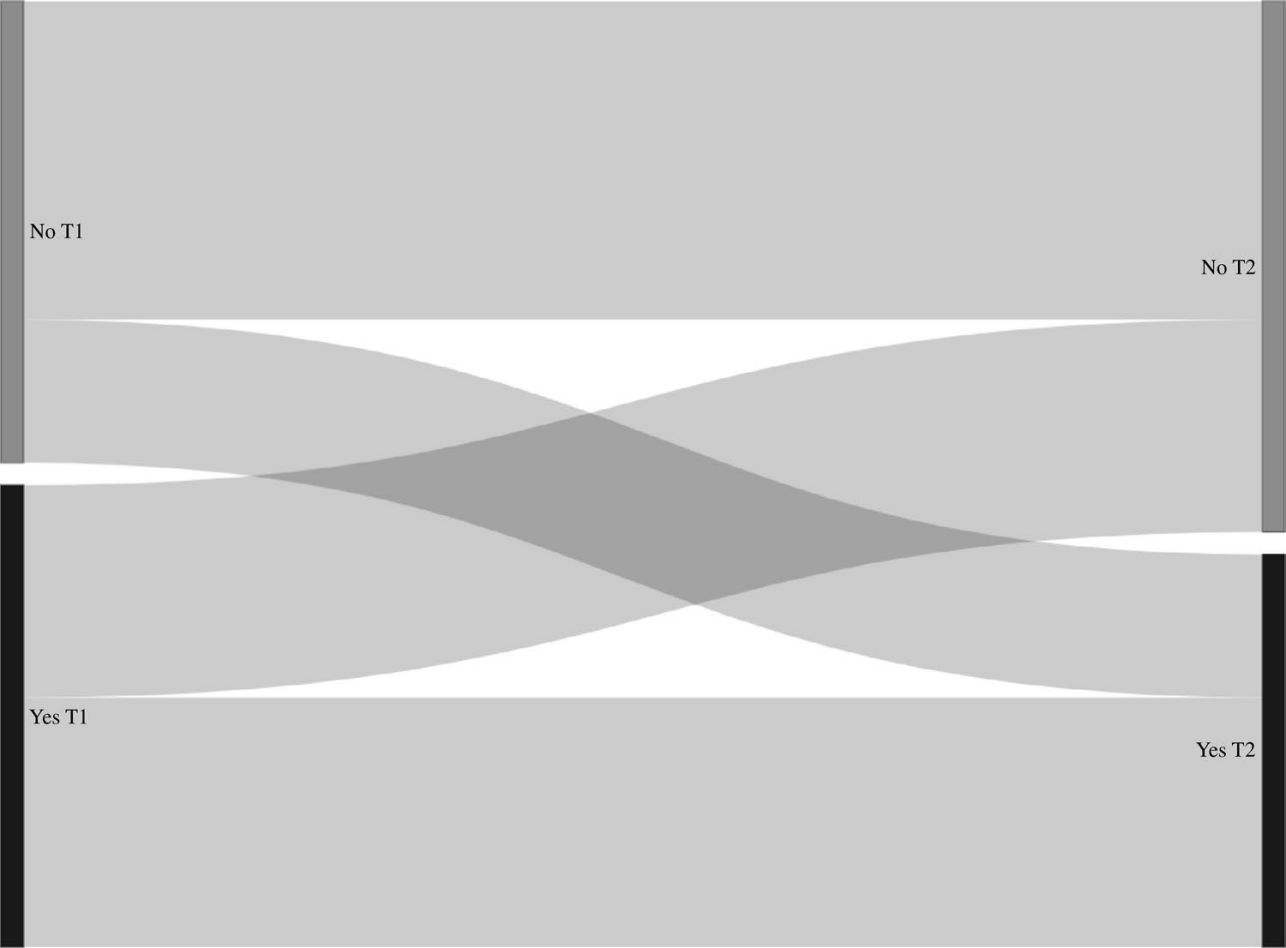
Transition probabilities for moving from one category at the current visit to another at the next visit for group sex

**Fig. 3:**
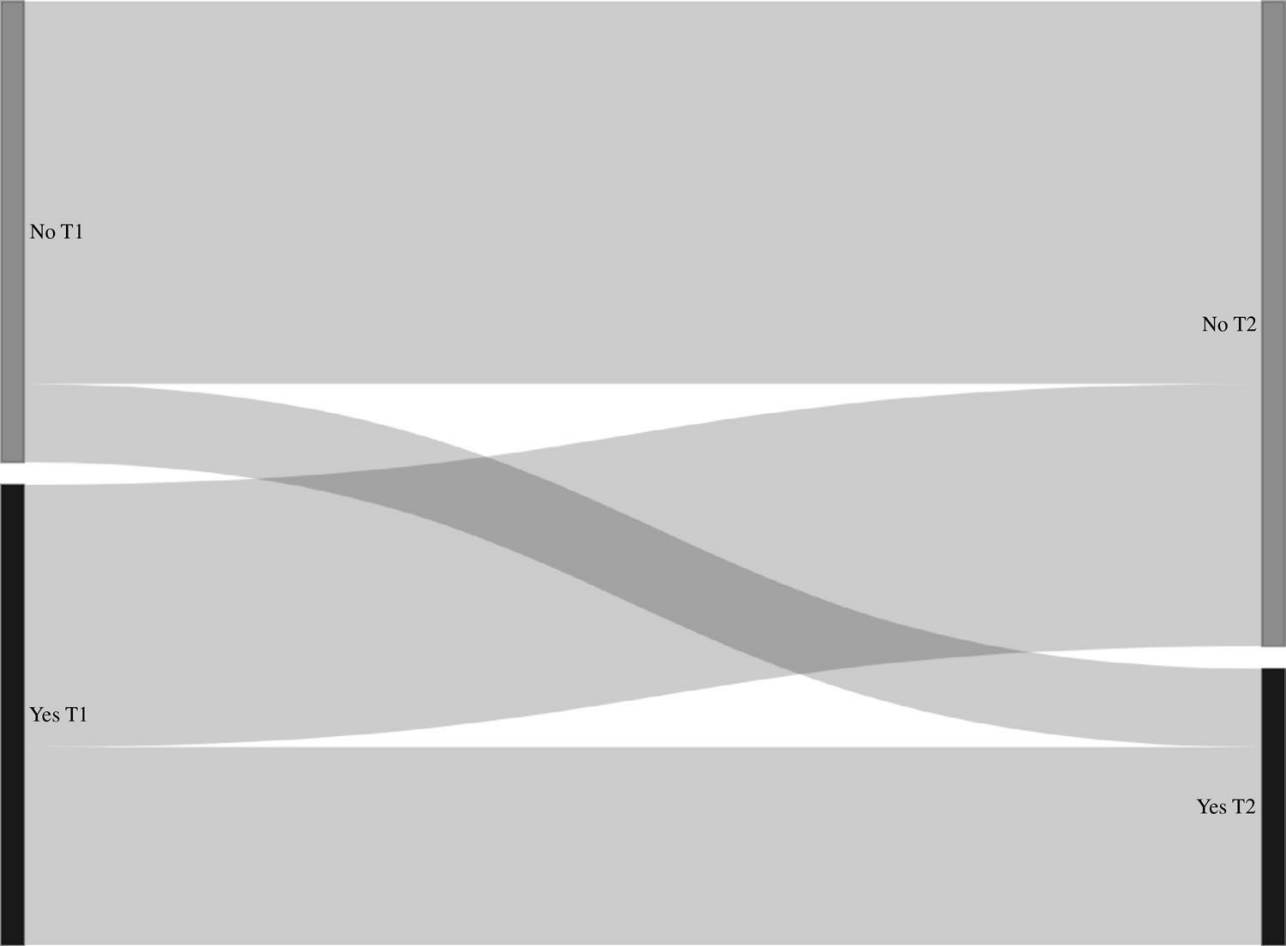
Transition probabilities for moving from one category at the current visit to another at the next visit for chemsex

**Fig. 4:**
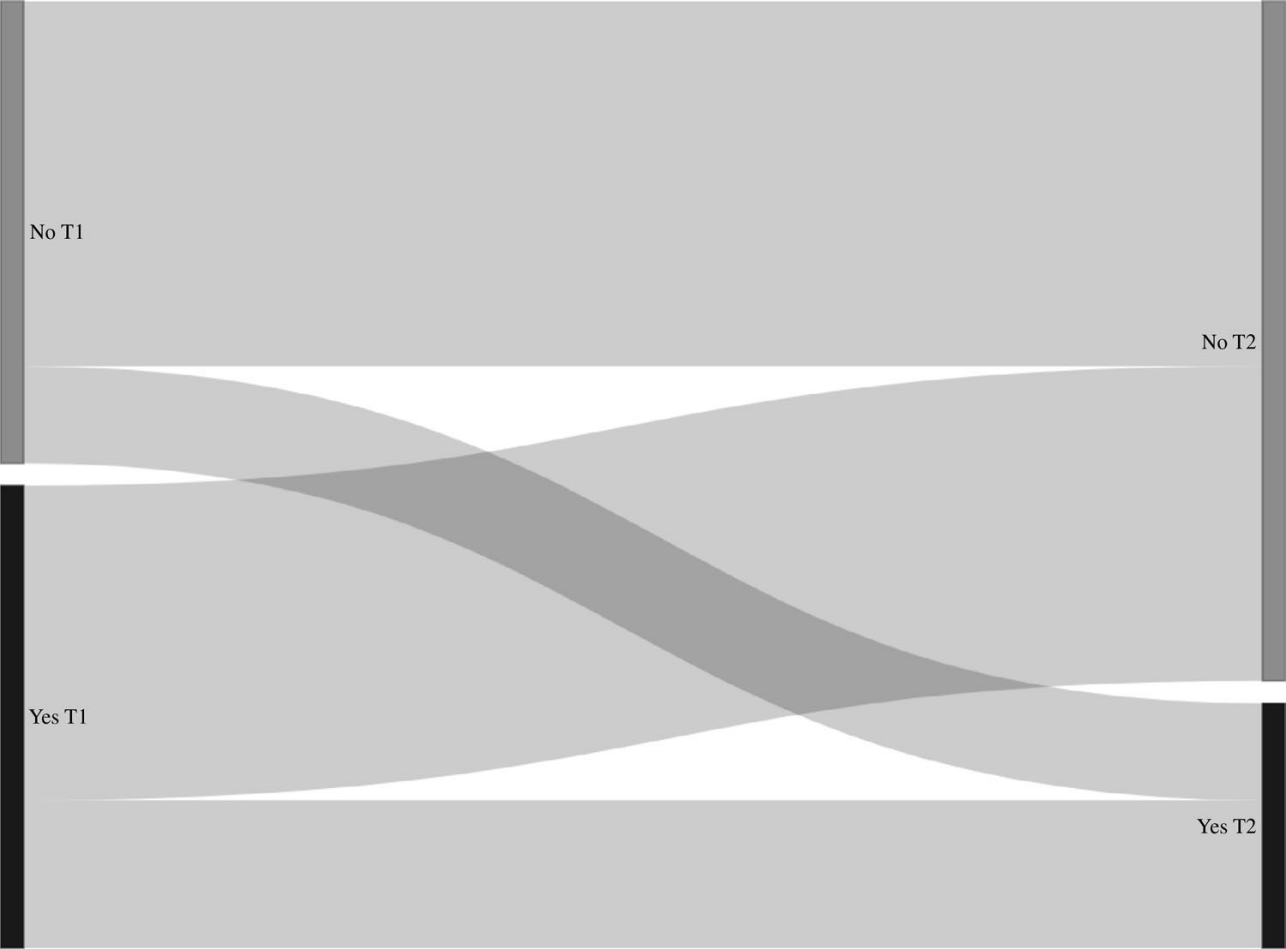
Transition probabilities for moving from one category at the current visit to another at the next visit for poppers and erection stimulants

**Fig. 5:**
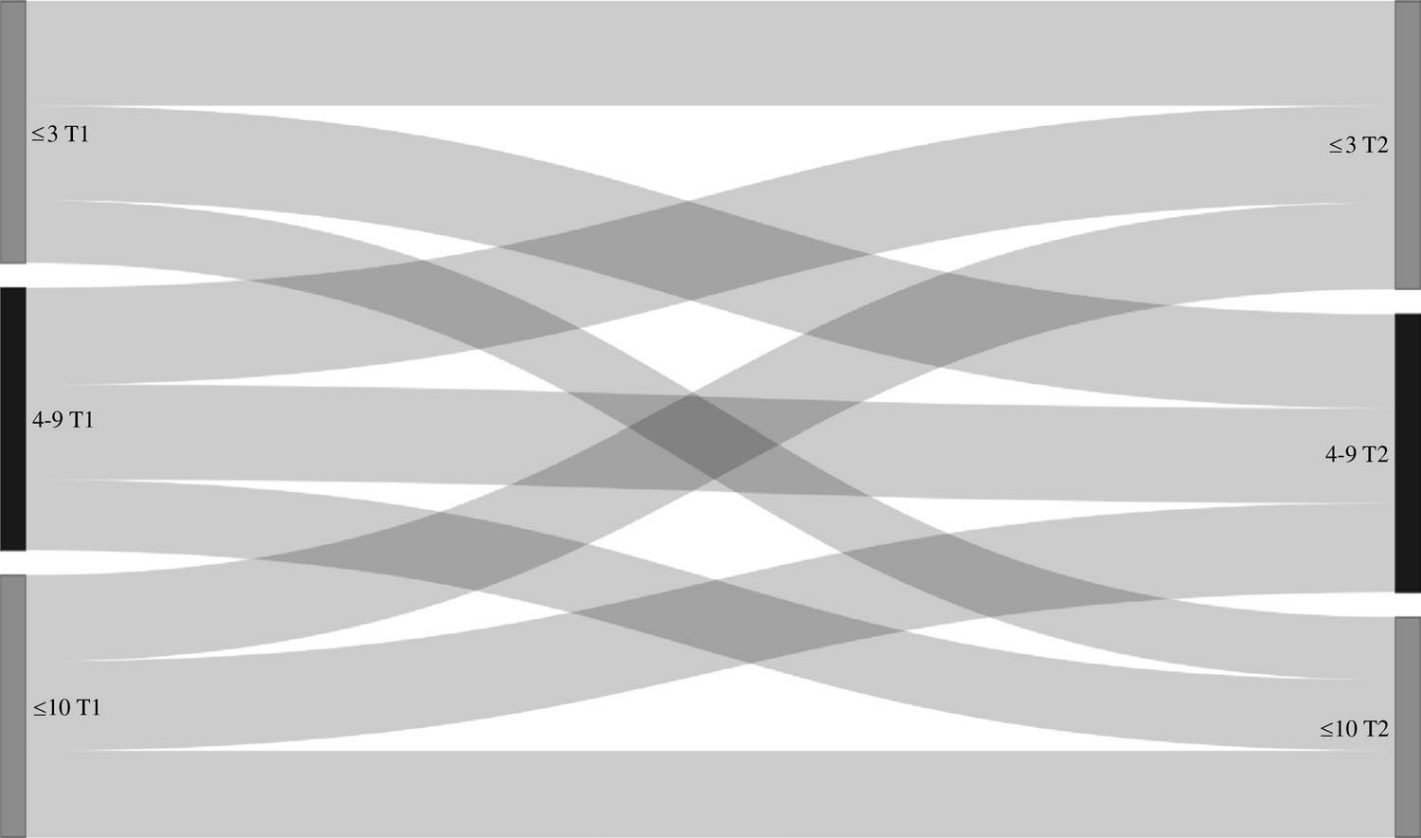
Transition probabilities for moving from one category at the current visit to another at the next visit for number of partners

**Fig. 6:**
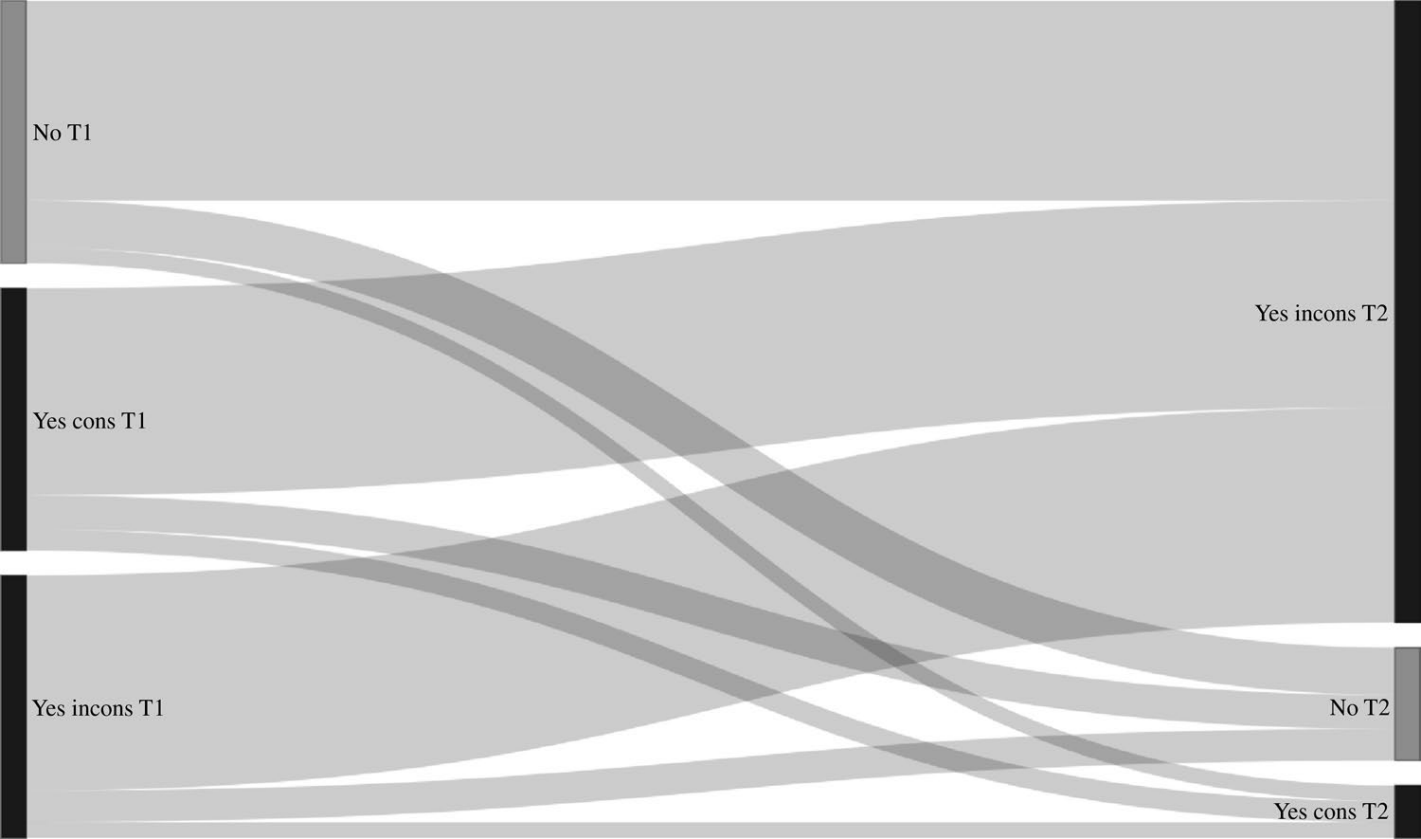
Transition probabilities for moving from one category at the current visit to another at the next visit for receptive anal sex. T1 = current visit; T2 = next visit; No = no insertive anal sex, Yes cons = Yes, consistent condom use, Yes incons = Yes, inconsistent condom use

**Fig. 7:**
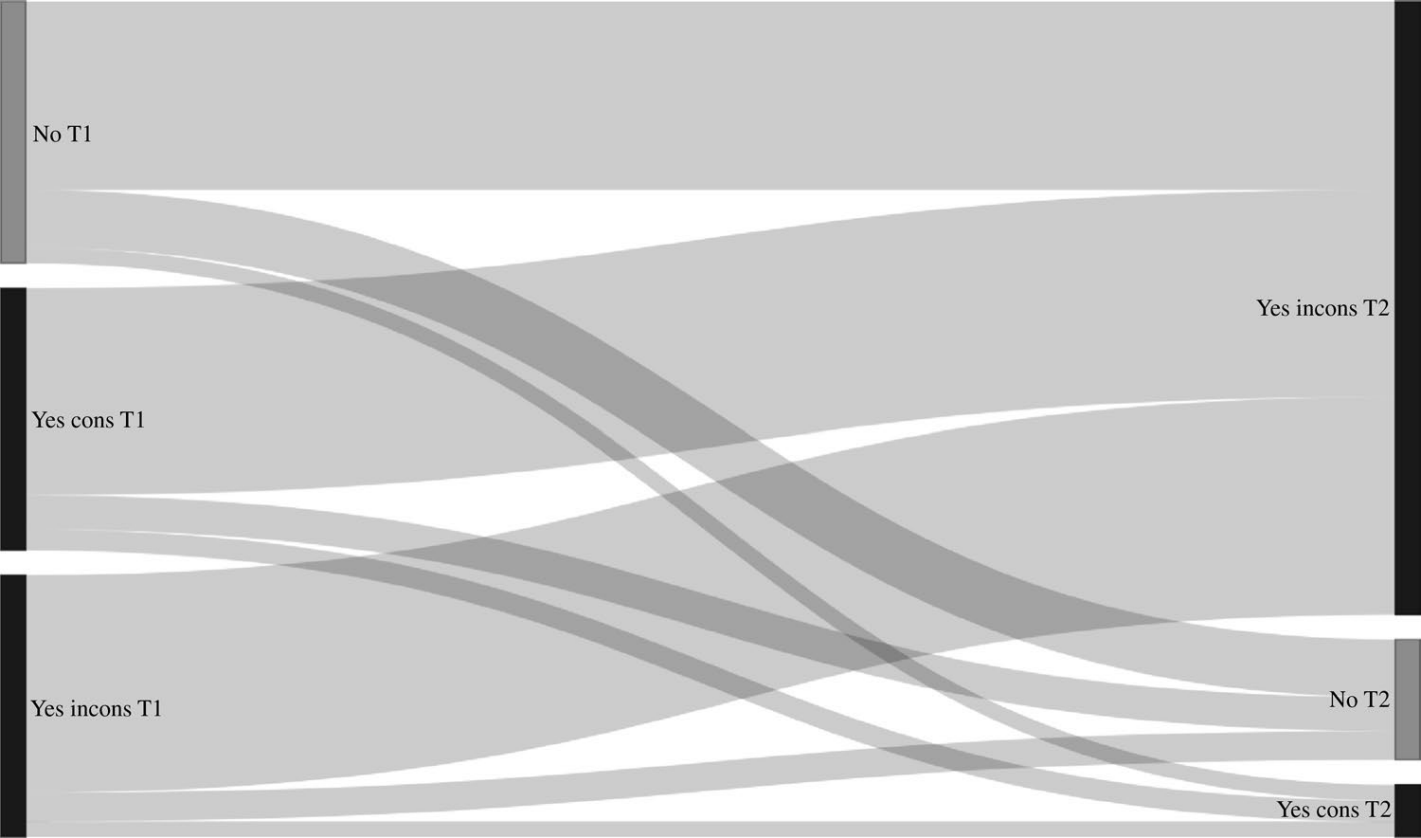
Transition probabilities for moving from one category at the current visit to another at the next visit for insertive anal sex T1 = current visit; T2 = next visit; No = no insertive anal sex, Yes cons = Yes, consistent condom use, Yes incons = Yes, inconsistent condom use

Transition probabilities derived from the Markov models for group sex (n = 2899, n visits = 11427), chemsex (n = 4328, n visits = 21173), poppers or erection stimulants (n = 4328, n visits = 21173), partner numbers (n = 4339, n visits = 21,630), receptive anal sex (n = 4318, n visits = 21223), and insertive anal sex (n = 4319, n visits = 21227) are shown in Table 3, and the corresponding transition matrix with 95% confidence intervals in Table S1. In these models, the probability of stopping group sex, chemsex, poppers or erection stimulants, and having a high partner numbers (i.e., transitioning from “yes” at the current visit to “no” at the next visit, or from “ ≥ 10 partners” to “ < 10 partners”) was higher compared to the probability of starting with these behaviors (i.e., transitioning from “no” at the current visit to “yes” at the next visit, or from “ < 10 partners” to “ ≥ 10 partners”) (Table 3, Figs. 2, 3, 4, 5, 6, and 7). Condom use among those reporting to have had receptive or insertive anal sex in the past 6 months decreased over time: consistent condom users had a high probability of changing to inconsistent condom use, and inconsistent condom users mostly remained inconsistent. When looking at the transition probabilities for specific time points, for all behaviors, we see that the probability of changing behavior increases over time (i.e., both stopping and starting with certain behaviors). However, at each time point (from first to second visit, from second to third, etcetera), the probability of stopping with certain behaviors is larger than the probability of starting with these behaviors over time (data not shown).

**Table 3:** Probability of transitioning from one category at the current SHC visit to another category at the next SHC visit in January 2018-June 2021 among men who have sex with men in the Dutch PrEP pilot

T1	T2		
Group sex			
	No	Yes	
No	0.69	0.31	
Yes	0.46	0.54	
Chemsex			
	No	Yes	
No	0.83	0.17	
Yes	0.57	0.43	
Poppers and erection stimulants			
	No	Yes	
No	0.79	0.21	
Yes	0.68	0.32	
Number of sex partners			
	≤ 3 partners	4–9 partners	≥ 10 partners
≤ 3 partners	0.40	0.36	0.24
4–9 partners	0.37	0.36	0.27
≥ 10 partners	0.33	0.34	0.33
Receptive anal sex			
	No	Yes, consistent condom use	Yes, inconsistent condom use
No	0.18	0.06	0.76
Yes, consistent condom use	0.13	0.08	0.79
Yes, inconsistent condom use	0.12	0.06	0.82
Insertive anal sex			
	No	Yes, consistent condom use	Yes, inconsistent condom use
No	0.22	0.06	0.72
Yes, consistent condom use	0.13	0.08	0.79
Yes, inconsistent condom use	0.11	0.06	0.83

*SHC* Sexual Health Centre *T1* Current visit *T2* Next visit

Reported behavior pertains to the past six months

### Predictors of Behavior Change

Anal STI or syphilis diagnosis at the current visit was a predictor of starting chemsex and use of poppers or erection stimulants, of continuing to engage in group sex and with inconsistent condom use, and of increasing partner numbers at the next visit (Tables 4, 5, 6, 7, 8, and 9). Non-anal STI diagnosis was a predictor of starting chemsex, and of a stable partner numbers (Tables 5, 7). First visit in the study period and visiting the SHC more often (vs. regular) were predictors of stopping with group sex, starting with chemsex and poppers or erection stimulants, and of increasing partner numbers the next visit (Tables 4, 5, 6, and 7). Furthermore, first visit was a predictor of changing to consistent condom use, whereas visiting the SHC more often (vs. regular) was a predictor of changing to inconsistent condom use (Tables 8, 9). Young participants (16–34 years) were more likely to stop with chemsex (Table 5), to change numbers of partners (increasing as well as decreasing) (Table 7), and to change from no sex or inconsistent condom use to anal sex with consistent condom use over time (Tables 8, 9).

**Table 4:** Univariable and multivariable determinants of changing behavior: Group sex (n = 2,899, n visits = 11,427)

	No—> Yes		Yes—> No	
	Crude	Adjusted	Crude	Adjusted
	HR (95% CI)	HR (95% CI)	HR (95% CI)	HR (95% CI)
Age > 34	1.00 (0.85–1.18)	1.06 (0.90–1.26)	0.90 (0.77–1.04)	0.95 (0.81–1.11)
Region, non-Amsterdam	**0.32 (0.25**–**0.42)**	**0.31 (0.24**–**0.41)**	**0.42 (0.33**–**0.52)**	**0.42 (0.33**–**0.53)**
Anal STI or syphilis diagnosis current visit	1.08 (0.87–1.34)	0.97 (0.78–1.21)	**0.75 (0.62**–**0.91)**	**0.70 (0.58**–**0.85)**
Non-anal STI diagnosis current visit	1.11 (0.79–1.57)	1.02 (0.72–1.46)	0.80 (0.58–1.10)	0.74 (0.53–1.02)
Time between consultations, (ref = regular)				
More often	1.14 (0.87–1.49)	0.92 (0.70–1.22)	0.97 (0.76–1.23)	0.85 (0.66–1.09)
Less often	0.97 (0.69–1.36)	0.94 (0.67–1.33)	0.89 (0.62–1.28)	0.87 (0.60–1.26)
First visit	**1.28 (1.06**–**1.54)**	0.98 (0.79–1.22)	**1.46 (1.24**–**1.73)**	**1.22 (1.00**–**1.47)**
COVID-19 (ref = Pre-COVID-19)				
Lockdown	**0.64 (0.50**–**0.81)**	**0.65 (0.49**–**0.85)**	**0.62 (0.50**–**0.77)**	**0.71 (0.56**–**0.90)**
Post-lockdown	**0.67 (0.54**–**0.83)**	**0.69 (0.52**–**0.90)**	**0.67 (0.54**–**0.83)**	**0.76 (0.60**–**0.97)**

Hazard ratios are shown in bold when the *p*-value < 0.05

*CI* Confidence Interval *HR* Hazard Ratio *STI* Sexually Transmitted Infection

**Table 5:** Univariable and multivariable determinants of changing behavior: Chemsex (n = 4,328, n visits = 21,173)

	No—> Yes		Yes—> No	
	Crude	Adjusted	Crude	Adjusted
	HR (95% CI)	HR (95% CI)	HR (95% CI)	HR (95% CI)
Age > 34	**0.79 (0.69**–**0.91)**	0.87 (0.76–1.00)	**0.76 (0.66**–**0.88)**	**0.82 (0.71**–**0.94)**
Region, non-Amsterdam	**0.55 (0.48**–**0.64)**	**0.56 (0.48**–**0.64)**	**0.40 (0.34**–**0.46)**	**0.40 (0.35**–**0.46)**
Anal STI or syphilis diagnosis current visit	**1.49 (1.26**–**1.76)**	**1.52 (1.29**–**1.80)**	0.91 (0.77–1.07)	0.90 (0.77–1.07)
Non-anal STI diagnosis current visit	**1.46 (1.12**–**1.89)**	**1.47 (1.13**–**1.91)**	0.95 (0.73–1.23)	0.88 (0.68–1.14)
Time between consultations, (ref = regular)				
More often	**1.53 (1.27**–**1.84)**	**1.41 (1.17**–**1.71)**	0.96 (0.79–1.15)	0.85 (0.71–1.03)
Less often	0.87 (0.68–1.12)	0.91 (0.70–1.18)	0.87 (0.68–1.11)	0.93 (0.72–1.19)
First visit	**1.29 (1.10**–**1.52)**	**1.20 (1.01**–**1.44)**	0.95 (0.79–1.13)	0.92 (0.76–1.11)
COVID-19 (ref = Pre-COVID-19)				
Lockdown	0.87 (0.74–1.03)	0.95 (0.80–1.13)	0.92 (0.78–1.09)	0.95 (0.80–1.14)
Post-lockdown	**0.82 (0.69**–**0.97)**	0.90 (0.75–1.08)	0.98 (0.83–1.17)	0.99 (0.83–1.18)

Hazard ratios are shown in bold when the *p*-value < 0.05

*CI* Confidence Interval *HR* Hazard Ratio *STI* Sexually Transmitted Infection

**Table 6:** Univariable and multivariable determinants of changing behavior: Poppers and erection stimulants (n = 4,328, n visits = 21,173)

	No—> Yes		Yes—> No	
	Crude	Adjusted	Crude	Adjusted
	HR (95% CI)	HR (95% CI)	HR (95% CI)	HR (95% CI)
Age > 34	0.96 (0.86–1.08)	1.08 (0.95–1.22)	0.93 (0.83–1.03)	1.02 (0.91–1.15)
Region, non-Amsterdam	**0.28 (0.24**–**0.31)**	**0.26 (0.23**–**0.30)**	**0.22 (0.20**–**0.25)**	**0.22 (0.20**–**0.25)**
Anal STI or syphilis diagnosis current visit	**1.20 (1.04**–**1.39)**	**1.31 (1.12**–**1.52)**	1.05 (0.92–1.19)	1.04 (0.91–1.20)
Non-anal STI diagnosis current visit	1.15 (0.92–1.45)	1.15 (0.90–1.46)	0.88 (0.70–1.11)	0.81 (0.63–1.03)
Time between consultations, (ref = regular)				
More often	**1.34 (1.14**–**1.57)**	1.16 (0.98–1.38)	**1.34 (1.16**–**1.54)**	1.08 (0.92–1.26)
Less often	0.90 (0.74–1.11)	1.00 (0.81–1.24)	1.04 (0.87–1.26)	1.01 (0.82–1.23)
First visit	**1.29 (1.13**–**1.48)**	**1.17 (1.00**–**1.37)**	**1.21 (1.06**–**1.38)**	1.07 (0.92–1.24)
COVID-19 (ref = Pre-COVID-19)				
Lockdown	**0.70 (0.61**–**0.80)**	**0.72 (0.62**–**0.84)**	**0.77 (0.68**–**0.88)**	0.92 (0.80–1.06)
Post-lockdown	**0.67 (0.58**–**0.77)**	**0.69 (0.59**–**0.81)**	**0.88 (0.77**–**1.00)**	0.97 (0.84–1.13)

Hazard ratios are shown in bold when the *p*-value < 0.05

*CI* Confidence Interval *HR* Hazard Ratio *STI* Sexually Transmitted Infections

**Table 7:** Univariable and multivariable determinants of changing behavior: Number of partners (n = 4,339, n visits = 21,630)

	≤ 3—> 4–9		≤ 3—> ≥ 10		4–9—> ≤ 3		4–9—> ≥ 10		≥ 10—> ≤ 3		≥ 10—> 4–9	
	Crude	Adjusted	Crude	Adjusted	Crude	Adjusted	Crude	Adjusted	Crude	Adjusted	Crude	Adjusted
	HR (95% CI)	HR (95% CI)	HR (95% CI)	HR (95% CI)	HR (95% CI)	HR (95% CI)	HR (95% CI)	HR (95% CI)	HR (95% CI)	HR (95% CI)	HR (95% CI)	HR (95% CI)
Age > 34	**0.82 (0.72**–**0.93)**	**0.84 (0.74**–**0.96)**	0.73 (0.48–1.11)	0.91 (0.59–1.40)	**0.84 (0.75**–**0.95)**	0.88 (0.78–1.00)	0.91 (0.78–1.05)	0.91 (0.79–1.06)	0.78 (0.61–1.01)	0.84 (0.64–1.11)	**0.79 (0.68**–**0.91)**	**0.77 (0.67**–**0.89)**
Region, non-Amsterdam	**0.67 (0.58**–**0.77)**	**0.71 (0.62**–**0.81)**	**0.53 (0.33**–**0.85)**	**0.49 (0.32**–**0.75)**	**0.50 (0.44**–**0.57)**	**0.55 (0.48**–**0.63)**	**0.58 (0.49**–**0.67)**	**0.59 (0.51**–**0.69)**	**0.43 (0.32**–**0.57)**	**0.32 (0.24**–**0.44)**	**0.68 (0.58**–**0.79)**	**0.76 (0.65**–**0.89)**
Anal STI or syphilis diagnosis current visit	0.92 (0.75–1.13)	0.92 (0.75–1.13)	**2.01 (1.29**–**3.14)**	**1.89 (1.17**–**3.04)**	1.03 (0.88–1.21)	1.01 (0.86–1.18)	0.92 (0.76–1.13)	0.95 (0.78–1.16)	0.92 (0.67–1.27)	1.02 (0.74–1.41)	0.97 (0.82–1.15)	0.93 (0.78–1.11)
Non-anal STI diagnosis current visit	0.95 (0.69–1.32)	0.86 (0.63–1.18)	1.31 (0.54–3.17)	1.54 (0.72–3.30)	**0.61 (0.45**–**0.81)**	**0.58 (0.43**–**0.79)**	0.98 (0.73–1.30)	0.97 (0.73–1.30)	1.16 (0.78–1.72)	1.05 (0.70–1.59)	0.84 (0.64–1.10)	0.84 (0.65–1.10)
Time between consultations, (ref = regular)												
More often	**1.47 (1.21**–**1.79)**	**1.33 (1.08**–**1.64)**	**5.59 (3.10**–**10.08)**	**4.53 (2.31**–**8.88)**	**1.24 (1.02**–**1.50)**	1.17 (0.95–1.43)	0.95 (0.74–1.20)	0.91 (0.71–1.16)	0.78 (0.49–1.23)	0.60 (0.32–1.11)	0.86 (0.70–1.06)	0.86 (0.70–1.06)
Less often	0.93 (0.75–1.15)	0.94 (0.76–1.17)	1.48 (0.65–3.37)	1.21 (0.45–3.23)	1.12 (0.94–1.33)	1.08 (0.90–1.31)	**0.70 (0.54**–**0.91)**	**0.73 (0.56**–**0.95)**	0.80 (0.48–1.33)	0.70 (0.38–1.28)	0.99 (0.80–1.23)	0.97 (0.78–1.23)
First visit	1.14 (0.97–1.35)	1.02 (0.85–1.22)	**2.72 (1.45**–**5.12)**	**2.11 (1.05**–**4.26)**	0.95 (0.82–1.10)	**0.83 (0.70**–**0.99)**	1.03 (0.87–1.22)	1.01 (0.84–1.21)	1.31 (0.99–1.73)	1.51 (1.08–2.12)	**0.73 (0.61**–**0.88)**	**0.66 (0.54**–**0.81)**
COVID-19 (ref = Pre-COVID-19)												
Lockdown	**0.82 (0.69**–**0.96)**	**0.81 (0.68**–**0.97)**	**0.31 (0.18**–**0.54)**	**0.41 (0.23**–**0.71)**	**0.80 (0.69**–**0.94)**	**0.77 (0.65**–**0.91)**	0.97 (0.81–1.16)	0.98 (0.81–1.18)	0.79 (0.58–1.07)	0.96 (0.67–1.37)	1.08 (0.91–1.29)	0.98 (0.82–1.18)
Post-lockdown	**0.75 (0.63**–**0.88)**	**0.75 (0.62**–**0.89)**	**0.37 (0.23**–**0.60)**	**0.46 (0.27**–**0.77)**	0.90 (0.77–1.04)	**0.82 (0.70**–**0.97)**	0.88 (0.73–1.06)	0.92 (0.75–1.11)	0.79 (0.57–1.09)	1.05 (0.72–1.52)	1.16 (0.97–1.38)	1.02 (0.85–1.22)

Hazard ratios are shown in bold when the *p*-value < 0.05

*CI* Confidence Interval *HR* Hazard Ratio *PrEP* Pre-exposure Prophylaxis *STI* Sexually Transmitted Infection

**Table 8:** Univariable and multivariable determinants of changing behavior: Receptive anal sex (n = 4,318, n visits = 21,223)

	No—> Yes, consistent condom use		No—> Yes, inconsistent condom use		Yes, consistent condom use—> No		Yes, consistent condom use—> Yes, inconsistent condom use		Yes, inconsistent condom use—> No		Yes, inconsistent condom use—> Yes, consistent condom use	
	Crude	Adjusted	Crude	Adjusted	Crude	Adjusted	Crude	Adjusted	Crude	Adjusted	Crude	Adjusted
	HR (95% CI)	HR (95% CI)	HR (95% CI)	HR (95% CI)	HR (95% CI)	HR (95% CI)	HR (95% CI)	HR (95% CI)	HR (95% CI)	HR (95% CI)	HR (95% CI)	HR (95% CI)
Age > 34	**0.42 (0.24**–**0.73)**	**0.45 (0.25**–**0.82)**	**0.74 (0.63**–**0.88)**	**0.77 (0.64**–**0.92)**	0.59 (0.33–1.06)	0.52 (0.26–1.01)	0.85 (0.72–1.01)	0.96 (0.79–1.15)	1.14 (0.98–1.32)	1.13 (0.96–1.32)	**0.46 (0.37**–**0.57)**	**0.48 (0.38**–**0.61)**
Region, non–Amsterdam	0.68 (0.36–1.27)	0.65 (0.34–1.25)	**0.53 (0.44**–**0.63)**	**0.51 (0.43**–**0.61)**	0.77 (0.39–1.52)	0.71 (0.35–1.44)	**0.49 (0.41**–**0.58)**	**0.50 (0.41**–**0.60)**	**0.49 (0.42**–**0.57)**	**0.48 (0.41**–**0.56)**	**0.50 (0.40**–**0.62)**	**0.51 (0.41**–**0.64)**
Anal STI or syphilis diagnosis current visit	1.36 (0.53–3.44)	1.22 (0.48–3.10)	1.10 (0.80–1.51)	1.09 (0.79–1.50)	0.62 (0.25–1.55)	0.67 (0.29–1.58)	1.01 (0.80–1.27)	1.01 (0.79–1.29)	**0.42 (0.32**–**0.54)**	**0.44 (0.34**–**0.57)**	**0.71 (0.54**–**0.94)**	**0.66 (0.49**–**0.89)**
Non-anal STI diagnosis current visit	1.08 (0.44–2.67)	–	0.82 (0.59–1.16)	–	**0.06 (0.01**–**3.08)**	–	0.75 (0.50–1.14)	–	1.18 (0.90–1.54)	–	0.72 (0.45–1.16)	–
Time between consultations, (ref = regular)												
More often	1.71 (0.70–4.16)	1.33 (0.48–3.70)	**1.31 (1.02**–**1.68)**	1.17 (0.90–1.51)	1.47 (0.69–3.10)	1.21 (0.50–2.92)	**1.42 (1.08**–**1.89)**	1.12 (0.83–1.51)	0.98 (0.79–1.21)	0.97 (0.77–1.21)	1.38 (0.99–1.91)	1.13 (0.80–1.61)
Less often	1.83 (0.76–4.43)	1.68 (0.63–4.47)	1.16 (0.87–1.55)	1.16 (0.86–1.56)	0.83 (0.34–2.03)	0.84 (0.33–2.10)	1.24 (0.95–1.61)	1.16 (0.87–1.53)	1.06 (0.84–1.33)	1.04 (0.81–1.32)	1.33 (0.93–1.90)	1.25 (0.86–1.83)
First visit	**2.39 (1.22**–**4.64)**	**2.10 (1.04**–**4.21)**	**1.34 (1.08**–**1.65)**	1.12 (0.89–1.41)	0.72 (0.37–1.40)	0.81 (0.39–1.68)	**1.27 (1.04**–**1.55)**	1.08 (0.86–1.35)	**0.80 (0.65**–**0.97)**	0.86 (0.67–1.07)	**2.14 (1.68**–**2.73)**	**1.87 (1.43**–**2.45)**
COVID-19 (ref = Pre-COVID-19)												
Lockdown	**0.44 (0.22**–**0.88)**	0.53 (0.23–1.21)	**0.46 (0.37**–**0.56)**	**0.44 (0.35**–**0.55)**	1.25 (0.63–2.46)	0.94 (0.46–1.93)	**0.72 (0.59**–**0.88)**	**0.78 (0.63**–**0.98)**	**0.78 (0.64**–**0.94)**	**0.76 (0.62**–**0.94)**	**0.63 (0.49**–**0.81)**	0.78 (0.59–1.03)
Post-lockdown	0.69 (0.33–1.44)	**0.88 (0.37**–**2.12)**	**0.73 (0.59**–**0.91)**	**0.71 (0.56**–**0.91)**	1.37 (0.67–2.83)	1.14 (0.54–2.41)	**0.80 (0.65**–**0.98)**	0.85 (0.67–1.07)	1.20 (1.00–1.45)	1.13 (0.92–1.39)	**0.72 (0.55**–**0.93)**	0.86 (0.65–1.15)

Hazard ratios are shown in bold when the *p*-value < 0.05

Non-anal STI diagnosis current visit was not included in this model, because the number of observations in the category ‘Yes, consistent condom use’ and a non-anal STI diagnosis was too small (< 5%)

*CI* Confidence Interval *HR* Hazard Ratio *PrEP* Pre-exposure Prophylaxis *STI* Sexually Transmitted Infection

**Table 9:** Univariable and multivariable determinants of changing behavior: Insertive anal sex (n = 4,319, n visits = 21,227)

	No—> Yes, consistent condom use		No—> Yes, inconsistent condom use		Yes, consistent condom use—> No		Yes, consistent condom use—> Yes, inconsistent condom use		Yes, inconsistent condom use—> No		Yes, inconsistent condom use—> Yes, consistent condom use	
	Crude	Adjusted	Crude	Adjusted	Crude	Adjusted	Crude	Adjusted	Crude	Adjusted	Crude	Adjusted
	HR (95% CI)	HR (95% CI)	HR (95% CI)	HR (95% CI)	HR (95% CI)	HR (95% CI)	HR (95% CI)	HR (95% CI)	HR (95% CI)	HR (95% CI)	HR (95% CI)	HR (95% CI)
Age > 34	0.72 (0.46–1.12)	0.73 (0.45–1.20)	**0.79 (0.67**–**0.93)**	0.87 (0.73–1.03)	**0.65 (0.43**–**0.98)**	0.75 (0.48–1.15)	0.91 (0.77–1.07)	0.95 (0.80–1.13)	**0.73 (0.61**–**0.86)**	**0.73 (0.61**–**0.87)**	**0.47 (0.38**–**0.58)**	**0.50 (0.40**–**0.63)**
Region, non–Amsterdam	0.62 (0.38–1.00)	0.75 (0.45–1.28)	**0.49 (0.42**–**0.58)**	**0.50 (0.42**–**0.59)**	**0.38 (0.26**–**0.57)**	**0.42 (0.27**–**0.63)**	**0.57 (0.48**–**0.67)**	**0.58 (0.49**–**0.69)**	**0.59 (0.49**–**0.70)**	**0.60 (0.50**–**0.71)**	**0.45 (0.36**–**0.55)**	**0.45 (0.36**–**0.56)**
Anal STI or syphilis diagnosis current visit	0.64 (0.32–1.25)	0.56 (0.27–1.15)	0.94 (0.76–1.17)	0.96 (0.77–1.19)	0.99 (0.57–1.72)	1.05 (0.59–1.84)	0.91 (0.72–1.15)	0.93 (0.73–1.19)	0.94 (0.76–1.17)	0.94 (0.75–1.18)	0.81 (0.61–1.06)	0.77 (0.58–1.02)
Non-anal STI diagnosis current visit	1.23 (0.44–3.44)	–	1.14 (0.75–1.73)	–	0.73 (0.30–1.76)	–	0.80 (0.57–1.12)	–	**0.65 (0.43**–**0.99)**	–	0.71 (0.45–1.11)	–
Time between consultations, (ref = regular)												
More often	**2.09 (1.01**–**4.35)**	1.48 (0.69–3.17)	1.21 (0.96–1.54)	1.11 (0.87–1.41)	1.02 (0.51–2.02)	0.78 (0.37–1.61)	**1.53 (1.20**–**1.95)**	**1.32 (1.02**–**1.71)**	0.83 (0.63–1.09)	**0.75 (0.56**–**0.99)**	**1.62 (1.19**–**2.21)**	1.36 (0.99–1.87)
Less often	1.39 (0.64–2.99)	1.18 (0.51–2.72)	0.85 (0.65–1.11)	0.85 (0.65–1.12)	0.83 (0.42–1.64)	0.82 (0.41–1.63)	1.10 (0.86–1.42)	1.07 (0.83–1.40)	1.20 (0.93–1.54)	1.12 (0.86–1.46)	1.23 (0.86–1.75)	1.21 (0.84–1.75)
First visit	**2.60 (1.54**–**4.39)**	**1.82 (1.00**–**3.32)**	0.89 (0.73–1.08)	0.87 (0.70–1.09)	1.11 (0.71–1.08)	0.89 (0.55–1.45)	1.03 (0.85–1.25)	0.95 (0.77–1.18)	1.06 (0.85–1.31)	0.94 (0.74–1.18)	**2.08 (1.63**–**2.65)**	1.93 (1.49–2.51)
COVID-19 (ref = Pre-COVID-19)												
Lockdown	**0.43 (0.25**–**0.74)**	**0.50 (0.27**–**0.93)**	0.97 (0.80–1.18)	0.97 (0.79–1.20)	**0.48 (0.28**–**0.82)**	**0.48 (0.27**–**0.85)**	0.84 (0.70–1.02)	0.84 (0.68–1.04)	0.76 (0.62––0.93)	0.76 (0.61–0.95)	0.62 (0.48–0.80)	0.77 (0.59–1.00)
Post-lockdown	**0.49 (0.28**–**0.84)**	0.59 (0.31–1.11)	0.95 (0.78–1.16)	0.93 (0.75–1.15)	0.83 (0.53–1.31)	0.80 (0.49–1.30)	0.94 (0.78–1.15)	0.93 (0.75–1.15)	0.82 (0.66–1.01)	0.80 (0.64–1.00)	0.73 (0.57–0.94)	0.88 (0.67–1.15)

Hazard ratios are shown in bold when the *p*-value < 0.05

Non-anal STI diagnosis current visit was not included in this model, because the number of observations in the category ‘No anal sex’ and a non-anal STI diagnosis was too small (< 5%)

*CI* Confidence Interval *HR* Hazard Ratio *PrEP* Pre-exposure Prophylaxis *STI* Sexually Transmitted Infection

Changes in group sex and condom use (i.e., both stopping or starting with the behaviors) were more likely pre-COVID-19 compared to during lockdown and post-lockdown periods (Tables 4, 8, 9). Furthermore, during lockdown and post-lockdown periods (vs pre-lockdown), participants were less likely to start with poppers or erection stimulants (Table 6), and to increase partner numbers (Table 7). Visiting the SHC in Amsterdam (vs. other regions) was a predictor of behavior change in general (i.e., both stopping or starting group sex, chemsex, poppers or erection stimulants, and condomless anal sex, and of changes in partner numbers). The AIC values for simpler models or with different Q-matrices were comparable to the AIC values of the final model, indicating appropriate goodness of fit.

## Discussion

Sexual behavior associated with increased likelihood of acquiring an STI, including group sex, chemsex, use of poppers or erection stimulants, high partner numbers (i.e., ≥ 10 partners), and condomless receptive or insertive anal sex, all in the past 6 months, was commonly reported by MSM in the Dutch national PrEP pilot between January 2018 and June 2021. These behaviors were relatively stable over time, however, MSM who did change their behavior over time were more likely to stop with group sex, chemsex, use of poppers or erection stimulants, and to decrease partner numbers, than to start with these behaviors after PrEP initiation, especially younger individuals (i.e., ≤ 34 years). In contrast, condom use during receptive or insertive anal sex decreased significantly over time, which was most pronounced among older individuals and individuals who received an STI diagnosis. Behavior change in general (i.e., stopping and starting) was more likely to occur pre-COVID-19 (i.e., often before PrEP initiation) and among individuals visiting the SHC in Amsterdam.

To our knowledge, this is the first study examining within-individual behavior change over time and identifying predictors of behavior change among PrEP users on a national level. We used extensive longitudinal data on STI/HIV test results, sexual behavior, and PrEP use from MSM in the Dutch national PrEP pilot. Furthermore, we were able to include a control period (i.e., SHC visits before initiation of PrEP), which may provide better insights into behavior change after PrEP initiation. There were also some limitations. First, data about group sex was missing for almost 50% of visits, because several SHC regions did not report this in 2019. This may have introduced biased results due to possible differences in study population. However, demographic characteristics and sexual behavior were not statistically different between visits with and without data on group sex (data not shown). Second, the frequency of the reported behaviors is unknown, which may be a predictor of behavior change, and an important indicator of exposure (i.e., likelihood of acquiring an STI). In addition, the behavioral variables included in the analysis pertained to the past 6 months, whereas visits were often every 3 months, which means that we might have overestimated the stability of behavior. Third, some categories of potential predictors were too small (< 5% of visits) to include them in the multivariable analyses, such as non-anal STI diagnoses. Similarly, some transition probabilities were small too (< 0.05), which in some models led to wide 95% confidence intervals. Last, the results might not be generalizable to all PrEP users in the Netherlands, as we did not include MSM who started PrEP before the PrEP pilot started, and MSM who used PrEP via another healthcare provider than the SHC. Nevertheless, the results help to understand trends in behavior in an extensive sample of PrEP users in the Netherlands.

We found that condom use decreased over time, a finding that previous studies among PrEP users also corroborate (Coyer et al., 2022; MacGregor et al., 2021; Zimmermann et al., 2021). Furthermore, those individuals who were already engaging in condomless anal sex were likely to continue doing so, similar to what has been found in another study among PrEP users in New Zealand (Saxton et al., 2022). Partner numbers in the past 6 months was highly variable over time, and we hypothesized that this could be due to COVID-19 since other studies found a reduced number of partners during COVID-19 compared to pre-COVID-19 among MSM (Hammoud et al., 2020; Rogers et al., 2022; van Bilsen et al., 2021). However, in our study, partner numbers were actually more stable during the COVID-19 pandemic. This inconsistency may be explained by differences in study population: MSM who stopped or decreased behaviors associated with increased likelihood of STI due to COVID-19 and/or did not use PrEP, as shown by these studies, were probably less likely to get an appointment at the SHC during COVID-19 (van Wees et al., 2022). Our study included MSM who still had PrEP and STI/HIV consultations (i.e., reported to have had condomless anal sex, and to be in need of PrEP pills) at the SHCs despite COVID-related restrictions, and downscaling of care. Furthermore, a study in Amsterdam among PrEP users showed that changes in partner numbers during lockdown were transient (Jongen et al., 2021a, 2021b). Since most of these studies only covered changes in the first lockdown period at the beginning of 2020, and we also included post-lockdown periods in 2020 and the lockdown in the first half of 2021, possible temporary reductions in partner numbers during the first lockdown may not be identifiable in the mean transition probabilities anymore. In addition, our study examined the reported partner numbers in the past 6 months, which means that a consultation during a lockdown period may still include months before COVID-19, or in post-lockdown periods. Moreover, it might be interesting to examine whether behaviors associated with increased likelihood of STI will increase after all COVID-19 restrictions have been lifted (i.e., from 2022 onwards).

STI or syphilis diagnosis at the current visit, first visit in the study period, and visiting the SHC more often (vs. regular) were predictors of continuing to engage in group sex at the next visit, of starting with chemsex and poppers or erection stimulants, and of increasing partner numbers. An explanation for this finding might be that chronologically MSM first start to engage in group sex, which increases the likelihood of acquiring an STI, and subsequently, start with chemsex and using poppers or erection stimulants, as these behaviors are associated with group sex (Sewell et al., 2017; Slurink et al., 2020). Furthermore, STI or syphilis diagnosis and first visit/visiting the SHC more often may also be indicators of starting a period of increased likelihood of acquiring an STI (Andresen et al., 2022; Basten et al., 2021). For example, individuals who visit the SHC more often than the recommended testing frequency, may be those who experienced STI-related symptoms or were notified for STI exposure (i.e., more likely to be diagnosed with STI). Furthermore, a previous study found among MSM found that STI diagnosis did not increase risk perception (Biello et al., 2019), which may explain the continuation of behaviors associated with increased likelihood of acquiring STI. Another explanation might be that MSM may have experienced reduced fear, shame and stigma after STI diagnosis and treatment, and may not feel inhibited anymore to engage in sexual behavior associated with increased likelihood of acquiring an STI and openly discuss their behavior during a consultation (Basten et al., 2021; Curley et al., 2022; Datta et al., 2019; van Wees et al., 2020; Zimmermann et al., 2021). However, the exact interaction between STI diagnosis, testing frequency, and behavior change remains unclear, and future research should focus on providing further insights into this association.

Although stable behavior was more common, we did observe a large shift towards stopping with group sex, chemsex, poppers or erection stimulants, and decreasing partner numbers over time. These results may indicate empowerment of MSM in terms of sexual decision-making. Results of previous studies also suggested that PrEP use may improve sexual well-being, such as increased self-esteem (Zimmermann et al., 2021), and decreased fear of acquiring HIV (Achterbergh et al., 2020). This may also have consequences for the likelihood of acquiring an STI, since these behaviors are often associated with condomless anal sex and STI transmission (Basten et al., 2021; De Baetselier et al., 2021; Evers et al., 2023; Flores Anato et al., 2022; Grov et al., 2013; Knoops et al., 2021; Knox et al., 2020; Maxwell et al., 2019; Prestage et al., 2009; Vosburgh et al., 2012). For example, even though condom use decreased over time, the partner numbers that participants had condomless sex with and the number of condomless sex acts may decrease, along with the likelihood of acquiring an STI. Furthermore, the increased frequency of PrEP follow-up consultations, and, thus, more exposure to behavioral counselling/motivational interviewing, may also positively influence behavior (Kumar et al., 2021; Starks et al., 2022).

Predictors of behavior change, such as age, and STI diagnoses, may help to identify MSM who are likely to start with or continue to engage in behaviors associated with increased likelihood of acquiring an STI in the near future. Subsequently, these predictors may be used to improve SHC consultations and provide proper counselling at the right time (i.e., before they change to behaviors associated with increased likelihood of acquiring STI/HIV) by means of harm reduction, which may reduce the likelihood of acquiring an STI among PrEP users and improve PrEP adherence. Since recent changes in national PrEP policy (2022) included optional decreased consultation frequency (e.g., every 4 or 6 months, instead of 3) on indication (Bierman et al., 2022), it might be interesting to evaluate the impact of this change on behavior trends in future research. Furthermore, another behavior, and potential predictors of this behavior, that might be interesting to explore over time in future research among PrEP users is PrEP adherence. Previous studies in the Netherlands showed that adherence to daily and event-driven PrEP based on tenofovir diphosphate levels measured with dried blood spots was generally high (Jongen et al., 2021a, 2021b; van den Elshout et al., 2023). They also found that several determinants, such as age and condomless anal sex with a casual partner, was associated with adherence, and that adherence may decrease over time (van den Elshout et al., 2023).

In conclusion, the results of this study provide better understanding of trends in sexual behavior among MSM in the Dutch national PrEP pilot. Sexual behavior associated with an increased likelihood of acquiring STI was more likely to decrease than increase between January 2018 and June 2021. However, inconsistent condom use increased significantly over time, especially among older individuals and individuals who received an STI diagnosis.

### Supplementary Information

Below is the link to the electronic supplementary material.

Supplementary file1 (DOCX 30 KB)

## Data Availability

This study used data from the Dutch national registration of sexual health centre consultations (SOAP). Pseudonymised individual participant data can be requested for scientific use with a methodologically sound proposal submitted to the SOAP registration committee for approval. Proposal forms and additional information can be requested via soap@ rivm.nl. Data requestors will need to sign a data access agreement.

## References

[CR1] Achterbergh RCA, Hoornenborg E, Boyd A, Coyer L, Meuzelaar SJA, Hogewoning AA, Davidovich U, van Rooijen MS, Schim van der Loeff MF, Prins M, de Vries HJC (2020). Changes in mental health and drug use among men who have sex with men using daily and event-driven pre-exposure prophylaxis: Results from a prospective demonstration project in Amsterdam, the Netherlands. EClinicalMedicine.

[CR2] Andresen S, Balakrishna S, Mugglin C, Schmidt AJ, Braun DL, Marzel A, Doco Lecompte T, Darling KE, Roth JA, Schmid P, Bernasconi E, Günthard HF, Rauch A, Kouyos RD, Salazar-Vizcaya L (2022). Unsupervised machine learning predicts future sexual behaviour and sexually transmitted infections among HIV-positive men who have sex with men. PLoS Computational Biology.

[CR3] Armstrong HL, Roth EA, Rich A, Lachowsky NJ, Cui Z, Sereda P, Card KG, Jollimore J, Howard T, Moore DM, Hogg RS (2018). Associations between sexual partner number and HIV risk behaviors: Implications for HIV prevention efforts in a treatment as prevention (TasP) environment. AIDS Care.

[CR4] Basten MGJ, van Wees DA, Matser A, Boyd A, Rozhnova G, den Daas C, Kretzschmar MEE, Heijne JCM (2021). Time for change: Transitions between HIV risk levels and determinants of behavior change in men who have sex with men. PLoS ONE.

[CR5] Biello KB, Edeza A, Montgomery MC, Almonte A, Chan PA (2019). Risk perception and interest in HIV pre-exposure prophylaxis among men who have sex with men with rectal gonorrhea and chlamydia infection. Archives of Sexual Behavior.

[CR6] Bierman, W., Hoornenborg, E., & Nellen, J. (2022). *Nederlandse multidisciplinaire richtlijn Pre-expositie profylaxe (PrEP) ter preventie van hiv (update 2022).* https://www.soaaids.nl/files/2022-07/20220711-PrEP-richtlijn-Nederland-versie-3-update-2022.pdf

[CR7] Coyer L, Prins M, Davidovich U, van Bilsen WPH, Schim van der Loeff MF, Hoornenborg E, Matser A, Boyd A (2022). Trends in sexual behavior and sexually transmitted infections after initiating human immunodeficiency virus pre-exposure prophylaxis in men who have sex with men from Amsterdam, the Netherlands: A longitudinal exposure-matched study. AIDS Patient Care and STDs.

[CR8] Curley CM, Rosen AO, Mistler CB, Eaton LA (2022). Pleasure and PrEP: A systematic review of studies examining pleasure, sexual satisfaction, and PrEP. Journal of Sex Research.

[CR9] Datta J, Reid D, Hughes G, Mercer CH, Wayal S, Weatherburn P (2019). Awareness of and attitudes to sexually transmissible infections among gay men and other men who have sex with men in England: A qualitative study. Sexual Health.

[CR10] De Baetselier I, Reyniers T, Platteau T, Wouters K, Nöstlinger C, Cuylaerts V, Buyze J, Laga M, Kenyon C, Crucitti T, Vuylsteke B (2021). Recurrent sexually transmitted infections among a cohort of men who have sex with men using pre-exposure prophylaxis in Belgium are highly associated with sexualized drug use. Sexually Transmitted Diseases.

[CR11] Evers YJ, Op den Camp KPL, Lenaers M, Dukers-Muijrers N, Hoebe C (2023). Alcohol and drug use during sex and its association with sexually transmitted infections: A retrospective cohort study among young people aged under 25 years visiting Dutch STI clinics. Sexually Transmitted Infections.

[CR12] Flores Anato JL, Panagiotoglou D, Greenwald ZR, Blanchette M, Trottier C, Vaziri M, Charest L, Szabo J, Thomas R, Maheu-Giroux M (2022). Chemsex and incidence of sexually transmitted infections among Canadian pre-exposure prophylaxis (PrEP) users in the l'Actuel PrEP Cohort (2013–2020). Sexually Transmitted Infections.

[CR13] Grov C, Rendina HJ, Ventuneac A, Parsons JT (2013). HIV risk in group sexual encounters: An event-level analysis from a national online survey of MSM in the US. Journal of Sexual Medicine.

[CR14] Hammoud MA, Maher L, Holt M, Degenhardt L, Jin F, Murphy D, Bavinton B, Grulich A, Lea T, Haire B (2020). Physical distancing due to COVID-19 disrupts sexual behaviors among gay and bisexual men in Australia: Implications for trends in HIV and other sexually transmissible infections. Journal of Acquired Immune Deficiency Syndromes.

[CR15] Hoornenborg, E., & Rijnders, B. (2019). HIV Pre-expositie profylaxe (PrEP) richtlijn Nederland. https://nvhb.nl/wp-content/uploads/2019/04/PrEP-richtlijn-Nederland-versie-2-dd-15-april-2019.pdf

[CR16] Hoornenborg E, Achterbergh RCA, Schim van der Loeff MF, Davidovich U, van der Helm JJ, Hogewoning A, van Duijnhoven YTHP, Sonder GJB, de Vries HJC, Prins M (2018). Men who have sex with men more often chose daily than event-driven use of pre-exposure prophylaxis: Baseline analysis of a demonstration study in Amsterdam. Journal of the International AIDS Society.

[CR17] Hoornenborg E, Coyer L, Achterbergh RCA, Matser A, Schim van der Loeff MF, Boyd A, van Duijnhoven YTHP, Bruisten S, Oostvogel P, Davidovich U (2019). Sexual behaviour and incidence of HIV and sexually transmitted infections among men who have sex with men using daily and event-driven pre-exposure prophylaxis in AMPrEP: 2 year results from a demonstration study. Lancet HIV.

[CR18] Hoornenborg E, Coyer L, van Laarhoven A, Achterbergh R, de Vries H, Prins M, Schim van der Loeff M (2018). Change in sexual risk behaviour after 6 months of pre-exposure prophylaxis use: Results from the Amsterdam Pre-exposure Prophylaxis Demonstration Project. AIDS.

[CR19] Jackson CH (2011). Multi-state models for panel data: The MSM package for R. Journal of Statistical Software.

[CR20] Jongen VW, Hoornenborg E, van den Elshout MA, Boyd A, Zimmermann HM, Coyer L, Davidovich U, Anderson PL, de Vries HJ, Prins M, Schim van der Loeff MF (2021). Adherence to event-driven HIV PrEP among men who have sex with men in Amsterdam, the Netherlands: Analysis based on online diary data, 3-monthly questionnaires and intracellular TFV-DP. Journal of the International AIDS Society.

[CR21] Jongen VW, Zimmermann HML, Boyd A, Hoornenborg E, van den Elshout MAM, Davidovich U, van Duijnhoven Y, de Vries HJC, Prins M, Schim van der Loeff MF, Coyer L (2021). Transient changes in preexposure prophylaxis use and daily sexual behavior after the implementation of COVID-19 restrictions among men who have sex with men. Journal of Acquired Immune Deficiency Syndromes.

[CR22] Kapland D (2008). An overview of Markov chain methods for the study of stage-sequential developmental processes. Developmental Psychology.

[CR23] Knoops, L., Poll van de, S., & Albers, T. (2021). *Slammen in Nederland*. Amsterdam

[CR24] Knox J, Boyd A, Matser A, Heijman T, Sandfort T, Davidovich U (2020). Types of group sex and their association with different sexual risk behaviors among HIV-negative men who have sex with men. Archives of Sexual Behavior.

[CR25] Kumar S, Haderxhanaj LT, Spicknall IH (2021). Reviewing PrEP's effect on STI incidence among men who have sex with men-balancing increased STI screening and potential behavioral sexual risk compensation. AIDS and Behavior.

[CR26] MacGregor L, Speare N, Nicholls J, Harryman L, Horwood J, Kesten JM, Lorenc A, Horner P, Edelman NL, Muir P, North P, Gompels M, Turner KME (2021). Evidence of changing sexual behaviours and clinical attendance patterns, alongside increasing diagnoses of STIs in MSM and TPSM. Sexually Transmitted Infections.

[CR27] Maxwell S, Shahmanesh M, Gafos M (2019). Chemsex behaviours among men who have sex with men: A systematic review of the literature. International Journal of Drug Policy.

[CR28] Prestage GP, Hudson J, Down I, Bradley J, Corrigan N, Hurley M, Grulich AE, McInnes D (2009). Gay men who engage in group sex are at increased risk of HIV infection and onward transmission. AIDS and Behavior.

[CR29] Prestage G, Maher L, Grulich A, Bourne A, Hammoud M, Vaccher S, Bavinton B, Holt M, Jin F (2019). Brief report: Changes in behavior after PrEP initiation among Australian gay and bisexual men. Journal of Acquired Immune Deficiency Syndromes.

[CR30] R Development Core Team. (2020). *R: A language and environment for statistical computing.* In (Version 4.2.0) R Foundation for Statistical Computing. https://www.r-project.org/

[CR31] Rijksoverheid. (2021). *Dashboard Coronavirus*. https://coronadashboard.rijksoverheid.nl/landelijk/maatregelen

[CR32] Rogers BG, Tao J, Darveau SC, Maynard M, Almonte A, Napoleon S, Murphy M, Chan PA (2022). The impact of COVID-19 on sexual behavior and psychosocial functioning in a clinical sample of men who have sex with men using HIV pre-exposure prophylaxis. AIDS and Behavior.

[CR33] Rozhnova G, Heijne JCM, Bezemer D, van Sighem A, Presanis A, De Angelis D, Kretzschmar MEE (2018). Elimination prospects of the Dutch HIV epidemic among men who have sex with men in the era of pre-exposure prophylaxis. AIDS.

[CR34] Sarno EL, Macapagal K, Newcomb ME (2021). "The main concern is HIV, everything else is fixable": Indifference toward sexually transmitted infections in the era of biomedical HIV prevention. AIDS and Behavior.

[CR35] Saxton PJW, Azariah S, Cavadino A, Forster RF, Jenkins R, Werder SF, Southey K, Rich JG (2022). Adherence, sexual behavior and sexually transmitted infections in a New Zealand prospective PrEP cohort: 12 months follow-up and ethnic disparities. AIDS and Behavior.

[CR36] Sewell J, Miltz A, Lampe FC, Cambiano V, Speakman A, Phillips AN, Stuart D, Gilson R, Asboe D, Nwokolo N, Clarke A, Collins S, Hart G, Elford J, Rodger AJ (2017). Poly drug use, chemsex drug use, and associations with sexual risk behaviour in HIV-negative men who have sex with men attending sexual health clinics. International Journal on Drug Policy.

[CR37] Slurink IAL, van Benthem BHB, van Rooijen MS, Achterbergh RCA, van Aar F (2020). Latent classes of sexual risk and corresponding STI and HIV positivity among MSM attending centres for sexual health in the Netherlands. Sexually Transmitted Infections.

[CR38] Smith DK, Pals SL, Herbst JH, Shinde S, Carey JW (2012). Development of a clinical screening index predictive of incident HIV infection among men who have sex with men in the United States. Journal of Acquired Immune Deficiency Syndromes.

[CR39] Starks TJ, Adebayo T, Kyre KD, Millar BM, Stratton MJ, Gandhi M, Ingersoll KS (2022). Pilot randomized controlled trial of motivational interviewing with sexual minority male couples to reduce drug use and sexual risk: The Couples Health Project. AIDS and Behavior.

[CR40] Statistics Netherlands (CBS). (2022). *New classification of population by origin*: *Replacing classification based on migration background and the concepts western/non-western*. Retrieved from https://www.cbs.nl/en-gb/longread/statistische-trends/2022/new-classification-of-population-by-origin

[CR41] Traeger MW, Schroeder SE, Wright EJ, Hellard ME, Cornelisse VJ, Doyle JS, Stoové MA (2018). Effects of pre-exposure prophylaxis for the prevention of human immunodeficiency virus infection on sexual risk behavior in men who have sex with men: A systematic review and meta-analysis. Clinical Infectious Diseases.

[CR42] van Bilsen WPH, Boyd A, van der Loeff MFS, Davidovich U, Hogewoning A, van der Hoek L, Prins M, Matser A (2020). Diverging trends in incidence of HIV versus other sexually transmitted infections in HIV-negative MSM in Amsterdam. AIDS.

[CR43] van Bilsen WPH, Zimmermann HML, Boyd A, Coyer L, van der Hoek L, Kootstra NA, Hoornenborg E, Prins M, Davidovich U, Matser A (2021). Sexual behavior and its determinants during COVID-19 restrictions among men who have sex with men in Amsterdam. Journal of Acquired Immune Deficiency Syndromes.

[CR44] van den Elshout MA, Hoornenborg E, Coyer L, Anderson PL, Davidovich U, de Vries HJ, Prins M, Schim van der Loeff MF (2023). Determinants of adherence to daily PrEP measured as intracellular tenofovir diphosphate concentrations over 24 months of follow-up among men who have sex with men. Sexually Transmitted Infections.

[CR45] van Wees DA, Drissen MCM, den Daas C, Heijman T, Kretzschmar MEE, Heijne JCM (2020). The impact of STI test results and face-to-face consultations on subsequent behaviour and psychological characteristics. Preventive Medicine.

[CR48] van Wees, D. A., Visser, M., van Aar, F., Op de Coul, E. L. M., Staritsky, L. E., Sarink, D., Willemstein, I. J. M., de Vries, A., Kusters, J. M. A., den Boogert, E., Alexiou, Z. W., Götz, H. M., Jansen, T., van Sighem, A. I., & Heijne, J. C. M. (2022). *Sexually transmitted infections in the Netherlands in 2021*. https://www.rivm.nl/publicaties/sexually-transmitted-infections-in-netherlands-in-2021

[CR46] Vosburgh HW, Mansergh G, Sullivan PS, Purcell DW (2012). A review of the literature on event-level substance use and sexual risk behavior among men who have sex with men. AIDS and Behavior.

[CR47] Walker ML, Stiasny D, Guy RJ, Law MG, Holt M, Mao L, Donovan B, Grulich AE, Gray RT, Regan DG (2022). Assessing the impact of HIV preexposure prophylaxis scale-up on gonorrhea incidence among gay and bisexual men in sydney: a mathematical modeling study. Sexually Transmitted Diseases.

[CR49] Zimmermann HML, Postma LR, Achterbergh RCA, Reyniers T, Schim van der Loeff MF, Prins M, de Vries HJC, Hoornenborg E, Davidovich U (2021). The impact of pre-exposure prophylaxis on sexual well-being among men who have sex with men. Archives of Sexual Behavior.

