# Supplementary material for: The Best Predictor of Future Behavior May Be the Past: Exploring Behavior Change in Men Who Have Sex with Men Using Pre-exposure Prophylaxis in the Netherlands
Source: Arch Sex Behav. 2024 May 6;53(7):2777–93. doi: 10.1007/s10508-024-02863-z (PMC11219441; doi:10.1007/s10508-024-02863-z)
Supplement: Supplementary file 1 — Supplementary file1 (DOCX 30 KB) [file 10508_2024_2863_MOESM1_ESM.docx]

**Supplementary Material**

**Table S1. Transition intensity matrix and 95% confidence intervals at baseline for MSM participating in the Dutch PrEP pilot**

|  | ***T2*** | |  |
| --- | --- | --- | --- |
| ***T1*** |  | |  |
| ***Groupsex*** | *No (TI (95%CI)* | *Yes (TI (95%CI)* |  |
| *No* | 0.55 (0.53-0.58) | 1.81 (1.72-1.90) |  |
| *Yes* | 2.43 (2.28-2.60) | 0.41 (0.38-0.44) |  |
| ***Chemsex*** | *No (TI (95%CI)* | *Yes (TI (95%CI)* |  |
| *No* | 0.74 (0.72-0.75) | 1.36 (1.33-1.39) |  |
| *Yes* | 2.81 (2.62-3.03) | 0.36 (0.33-0.38) |  |
| ***Poppers and erection stimulants*** | *No (TI (95%CI)* | *Yes (TI (95%CI)* |  |
| *No* | 0.58 (0.56-0.60) | 1.72 (1.67-1.77) |  |
| *Yes* | 5.53 (5.05-6.07) | 0.18 (0.16-0.20) |  |
| ***Number of sex partners*** | *≤3 partners (TI (95%CI)* | *4-9 partners (TI (95%CI)* | *≥10 partners (TI (95%CI)* |
| *≤3 partners* | 0.10 (0.09-0.12) | 7.11 (6.29-8.10) | 1.34 (1.27-8.1.43) |
| *4-9 partners* | 7.10 (6.34-8.00) | 0.04 (0.04-0.05) | 3.20 (2.95-3.49) |
| *≥10 partners* | 1.71 (1.60-1.84) | 3.90 (3.55-4.30) | 0.15 (0.14-0.16) |
| ***Receptive anal sex*** | *No (TI (95%CI)* | *Yes, consistent condom use (TI (95%CI)* | *Yes, inconsistent condom use (TI (95%CI)* |
| *No* | 0.09 (0.07-0.10) | 1.38 (1.27-1.53) | 8.27 (6.96-9.98) |
| *Yes, consistent condom use* | 1.60 (1.43-1.85) | 0.03 (0.02-0.04) | 22.5 (17.5-29.6) |
| *Yes, inconsistent condom use* | 1.43 (1.39-1.47) | 1.24 (1.21-1.26) | 0.57 (0.55-0.59) |
| ***Insertive anal sex*** | *No (TI (95%CI)* | *Yes, consistent condom use (TI (95%CI)* | *Yes, inconsistent condom use (TI (95%CI)* |
| *No* | 0.14 (0.12-0.16) | 1.43 (1.33-1.56) | 5.07 (4.47-5.80) |
| *Yes, consistent condom use* | 1.97 (1.75-2.28) | 0.02 (0.02-0.03) | 21.8 (17.2-28.2) |
| *Yes, inconsistent condom use* | 1.29 (1.27-1.32) | 1.25 (1.22-1.28) | 0.62 (0.60-0.64) |

Abbreviations: SHC=Sexual Health Centre; T1=Current visit; T2=Next visit
Footnote: Reported behavior pertains to the past six months.
